# Use of cyclosporine in children and adolescents with moderate to severe atopic dermatitis: clinical experience in a tertiary Hospital^⋆^

**DOI:** 10.1016/j.abd.2022.10.006

**Published:** 2023-03-25

**Authors:** Carolina Contin Proença, Silvia Assumpção Soutto Mayor, Thais Storni Ragazzo, Sandy Daniele Germano Munhoz, Carolina Gadelha Pires

**Affiliations:** Dermatology Clinic, Santa Casa de São Paulo, São Paulo, SP, Brazil

Dear Editor,

Atopic dermatitis (AD) is one of the most common dermatoses in the pediatric age group, and its prevalence has increased in recent years worldwide.^1^ In a systematic review, the prevalence reached 22.6% in children and up to 17% in adolescents and adults.^2^

A dysfunctional skin barrier, an inflammatory response with a predominance of the Th2 pattern and altered microbiome are part of the disease etiopathogenesis. Bacterial and viral infections are frequent and the use of systemic immunosuppressants is necessary in moderate and severe cases.^3^

AD severity can be measured using scales such as SCORAD (Severity Scoring of Atopic Dermatitis Index). A score of 0 to 28 corresponds to mild AD; 29 to 48 to moderate AD; and 49 to 103 to severe AD.^4^ A positive association between AD and severe depression in adolescents has been described. Post-treatment clinical improvement seems to reduce this risk.^5^

Systemic corticosteroids, cyclosporine A, methotrexate, phototherapy, and measures to restore the skin barrier, such as moisturizers and emollients are used in the management of severe cases.^1^, ^3^ Cyclosporine is approved in many European countries and in Brazil to treat severe AD in adults. It can be used in children and adolescents with severe and refractory AD (off-label).^5^ The dose used is 3 to 5 mg/kg/day. There are reports in the literature of starting doses of up to 7 mg/kg/day, gradually reduced to 3 mg/kg/day (maintenance dose) for periods of up to two years.^1^, ^6^

In Brazil, the National Health Surveillance Agency (ANVISA, *Agência Nacional de Vigilância Sanitária*) has approved the use of cyclosporine for treatment of severe atopic dermatitis in adults, but it is not available at the Brazilian Unified Health System (SUS, *Sistema Único de Saúde*), which hinders access to this drug.

The authors carried out a retrospective observational study by reviewing the medical records and photographic records of 16 patients aged between five and 19 years (mean age: 11.94 years, standard deviation of 4.37, and median of 12 years), diagnosed with moderate to severe AD, followed at the Pediatric Dermatology Outpatient Clinic of a referral tertiary hospital in the city of São Paulo, Brazil, between 2011 and 2021.

The patients included in the study received cyclosporine A, on average 3 mg/kg/day, for a minimum period of six months. Therapeutic response, drug side effects, and non-atopic pre-existing comorbidities, such as depression, were assessed.

Sixteen patients were included: 9 (56.3%) female and 7 (43.8%) male individuals. Fourteen (87.5%) had been admitted to the hospital at least once and 6.3% were admitted six times due to secondary bacterial and viral skin infections, which could be related to treatment with cyclosporine.

Table 1 depicts the pre-and post-treatment SCORAD scores with cyclosporine treatment, showing a significant decrease in the SCORAD scores after treatment (p = 0.001).

**Table 1:** Pre- and post-treatment SCORAD descriptive values.

Moment	n	Mean	SD	Minimum	Maximum
Pre	16	69.54	12.10	50.00	95.00
Post	16	38.25	13.28	22.10	79.55

p* = 0.001 (*) Descriptive level of probability of Wilcoxon non-parametric test.

Fig. 1 shows the SCORAD mean and standard deviation values before and after the use of cyclosporine. Treatment duration ranged from one to 30 months (mean of 11.73 months with a standard deviation of 7.06 months and median of 11 months). Three patients (18.8%) discontinued treatment: one due to bacterial skin infection, one due to a liver transplant because of autoimmune liver disease, unrelated to cyclosporine use, and another for an unknown reason. Seven patients (43.8%) had side effects: four (25%) had hypertrichosis and three (18.7%) had bacterial and viral skin infections. Potentially severe adverse effects, such as increased serum creatinine and isolated arterial hypertension, more common in adults,^7^, ^8^ were not observed in the present study.

**Figure 1:**
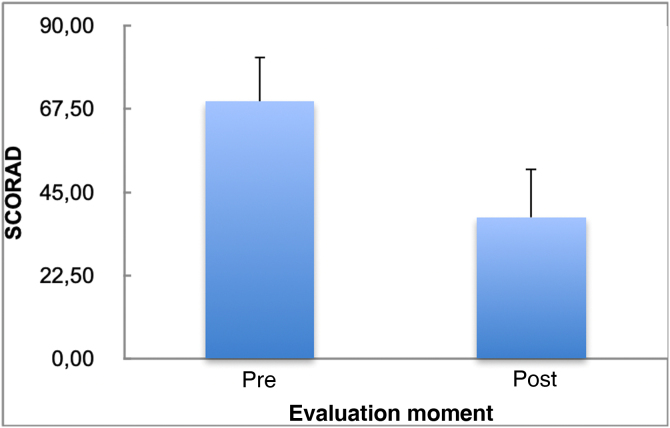
SCORAD mean and standard deviation values in the pre- and post-treatment moments.

Harper et al. described transient alterations in serum creatinine levels in four children, which regressed spontaneously or soon after the reduction in the cyclosporine dose. Although side effects are more common in adults, monitoring of blood pressure and renal function should be performed when prescribing cyclosporine to the pediatric population.^6^, ^7^

The SCORAD percentage variation delta (post value – pre value/pre value*100) ranges from -76.11% to 11.18% (mean of -43.71% with a standard deviation value of 19.95% and median of -49.28%).

There was a reduction of more than 30% in SCORAD scores in 75% of patients treated with cyclosporine A, which demonstrates therapeutic efficacy. Similar data have been reported in the literature.^7^, ^9^, ^10^

There was no significant correlation between treatment duration and the SCORAD variation delta (Spearman correlation coefficient: r = 0.035; p = 0.896).

The groups with and without side effects did not show a significant difference in relation to the SCORAD variation delta.

The occurrence of malignant neoplasms (lymphomas and squamous cell carcinoma), may be associated with the use of high doses of cyclosporine in transplant patients, and also with the use of cyclosporine in patients with psoriasis that were previously treated with methotrexate and/or phototherapy.^8^ No case of malignant neoplasm, associated with the use of cyclosporine, was observed in this study or any other similar in the literature.^8^

Among the comorbidities present before treatment with cyclosporine, depression, diagnosed by a psychiatrist, was found in 43.8% of the patients.

Although more frequent in adult patients with moderate and severe AD, children and adolescents with atopic dermatitis have shown severe depression and other psychiatric disorders.^5^ More studies with the pediatric population are required to elucidate the correlation between psychiatric diseases and atopic dermatitis.

The use of cyclosporine A in the treatment of moderate and severe forms of AD in adults is effective and well-documented in the literature. There is a lack of studies on the pediatric population in Brazil.

The present study allowed the authors to conclude that treatment with cyclosporine was effective and well tolerated by children and adolescents with moderate/severe AD, at a dose of 3 to 5 mg/Kg/day.

## Financial support

None declared.

## Authors' contributions

Carolina Contin Proença: Approval of the final version of the manuscript; drafting and editing of the manuscript; collection, analysis, and interpretation of data; effective participation in research orientation; intellectual participation in the propaedeutic and/or therapeutic conduct of the studied cases; critical review of the literature; critical review of the manuscript.

Silvia Assumpção Mayor: Approval of the final version of the manuscript; design and planning of the study; collection, analysis, and interpretation of data; effective participation in research orientation; intellectual participation in the propaedeutic and/or therapeutic conduct of the studied cases; critical review of the literature; critical review of the manuscript.

Thaiz Storni Ragazzo: Approval of the final version of the manuscript; design and planning of the study; collection, analysis, and interpretation of data; intellectual participation in the propaedeutic and/or therapeutic conduct of the studied cases; critical review of the literature; critical review of the manuscript.

Sandy Daniele Germano Munhoz: Approval of the final version of the manuscript; design and planning of the study; collection, analysis, and interpretation of data; intellectual participation in the propaedeutic and/or therapeutic conduct of the studied cases; critical review of the literature; critical review of the manuscript.

Carolina Gadelha Pires: Approval of the final version of the manuscript; design and planning of the study; collection, analysis, and interpretation of data; intellectual participation in the propaedeutic and/or therapeutic conduct of the studied cases; critical review of the literature; critical review of the manuscript.

## Conflicts of interest

None declared.
